# Dysfunctions of the basal ganglia-cerebellar-thalamo-cortical system produce motor tics in Tourette syndrome

**DOI:** 10.1371/journal.pcbi.1005395

**Published:** 2017-03-30

**Authors:** Daniele Caligiore, Francesco Mannella, Michael A. Arbib, Gianluca Baldassarre

**Affiliations:** 1 Laboratory of Computational Embodied Neuroscience, Institute of Cognitive Sciences and Technologies, National Research Council (CNR-ISTC-LOCEN), Roma, Italy; 2 Neuroscience Program, USC Brain Project, Computer Science Department, University of Southern California, Los Angeles, California, United States of America; Johns Hopkins University, UNITED STATES

## Abstract

Motor tics are a cardinal feature of Tourette syndrome and are traditionally associated with an excess of striatal dopamine in the basal ganglia. Recent evidence increasingly supports a more articulated view where cerebellum and cortex, working closely in concert with basal ganglia, are also involved in tic production. Building on such evidence, this article proposes a computational model of the basal ganglia-cerebellar-thalamo-cortical system to study how motor tics are generated in Tourette syndrome. In particular, the model: (i) reproduces the main results of recent experiments about the involvement of the basal ganglia-cerebellar-thalamo-cortical system in tic generation; (ii) suggests an explanation of the system-level mechanisms underlying motor tic production: in this respect, the model predicts that the interplay between dopaminergic signal and cortical activity contributes to triggering the tic event and that the recently discovered basal ganglia-cerebellar anatomical pathway may support the involvement of the cerebellum in tic production; (iii) furnishes predictions on the amount of tics generated when striatal dopamine increases and when the cortex is externally stimulated. These predictions could be important in identifying new brain target areas for future therapies. Finally, the model represents the first computational attempt to study the role of the recently discovered basal ganglia-cerebellar anatomical links. Studying this non-cortex-mediated basal ganglia-cerebellar interaction could radically change our perspective about how these areas interact with each other and with the cortex. Overall, the model also shows the utility of casting Tourette syndrome within a system-level perspective rather than viewing it as related to the dysfunction of a single brain area.

## Author summary

Tourette syndrome is a neuropsychiatric disorder characterized by vocal and motor tics. Tics represent a cardinal symptom traditionally associated with a dysfunction of the basal ganglia leading to an excess of the dopamine neurotransmitter. This view gives a restricted clinical picture and limits therapeutic approaches because it ignores the influence of altered interactions between the basal ganglia and other brain areas. In this respect, recent evidence supports a more articulated framework where cerebellum and cortex are also involved in tic production. Building on these data, we propose a computational model of the basal ganglia-cerebellar-thalamo-cortical network to investigate the specific mechanisms underlying motor tic production. The model reproduces the results of recent experiments and suggests an explanation of the system-level processes underlying tic production. Moreover, it furnishes predictions related to the amount of tics generated when there are dysfunctions in the basal ganglia-cerebellar-thalamo-cortical circuits. These predictions could be important in identifying new brain target areas for future therapies based on a system-level view of Tourette syndrome.

## Introduction

Tourette syndrome (TS) is a neuropsychiatric disorder characterized by the presence of sudden and repetitive involuntary movements or vocalizations, generally termed as “tics”, having differing degrees of intensity and frequency, and unpredictable duration [1, 2]. Tics can be simple, for example involving eye blinking, facial grimacing, shoulder shrugging, sniffing, or complex, involving more elaborated manifestations like touching objects, clapping, obscene gestures, or repetition of words [3, 4]. The typical age of onset of TS is around five to seven years and the course of the disease can be quite variable. In addition to tics, children with TS can show a variety of comorbid psychopathologies, including learning difficulties, sleep abnormalities, anxiety, obsessive-compulsive disorder (OCD), and attention deficit hyperactivity disorder (ADHD) [5, 6] (see S1 Table in the Supporting Information for all the main abbreviations used in the article). Usually, most TS symptoms decline during adolescence or early adulthood [7].

Motor tics are a cardinal symptom of TS shared with several neurological impairments including dystonia [8], Huntington’s disease [9, 10] and OCD [11, 12]. Traditionally, tics in TS are associated with basal ganglia abnormalities and in particular with a dysfunction of the striatal GABAergic networks leading to an excess of striatal dopamine [13–16]. This excess might cause an abnormal functioning of the basal ganglia-thalamo-cortical circuit leading to the production of tics [17]. To understand how this circuit may operate in TS, we first briefly describe how it typically works in healthy subjects (see section “The basal ganglia and their loops with the thalamo-cortical system: anatomy and physiology” for more details). In general, the basal ganglia promote movement generation of some specific motor patterns within primary motor cortex via a double-inhibition mechanism while maintaining tonic inhibitory control over other patterns [18–21]. In non-pathological conditions, the inhibition of specific GABAergic output nuclei of the basal ganglia leads to release the activity within the target thalamus areas forming loops with primary motor cortex, thus allowing the focused disinhibition of specific motor patterns. The basal-ganglia double-inhibition mechanism also targets sub-cortical areas, although in this case without the mediation of the thalamus, for example the superior colliculus for eye movements [22, 23]. An alteration in striatal dopamine release as in TS may induce the production of tics as a consequence of a focal excitatory abnormality in the striatum that causes an undesired disinhibition of thalamo-cortical circuits [15, 17] whose effect is the production of tics.

The basal ganglia are strongly linked, both anatomically and functionally, with several cortical regions and with the cerebellum. The basal ganglia and cerebellum receive input from, and send output to, cortex through multisynaptic anatomically partially segregated loops performing distinct functional operations within the motor and cognitive realms [24–27]. Studies in rats [28] and monkeys [29] have demonstrated that the cerebellum has a strong disynaptic projection to the striatum mediated by the intralaminar nuclei of the thalamus. Complementary to this, recent investigations on monkeys have shown that the subthalamic nucleus, an important component of the basal ganglia, has a disynaptic projection to the cerebellar cortex by way of the pontine nuclei [30]. Similar data have been found in humans [31]. These data have stimulated new research to investigate the role of the cerebellum and basal ganglia in functions typically associated with cortex (e.g., action understanding, [32–35]), and the involvement of cortical and cerebellar regions in impairments typically associated with basal ganglia such as Parkinson’s disease [36–46] and TS [47–49].

This *system-level perspective* [50, 51], according to which the basal ganglia work in concert with cortex and cerebellum to produce motor and cognitive behaviours of various complexity [26, 35, 52–55], renders the whole picture of TS pathophysiology more complex [56]. In particular, the specific contribution of cerebellar and cortical areas to basal ganglia-mediated tic expression remains unknown. The cerebellar activation found in several studies on tics may reflect an increase of afferent sensory input driven by overt tic movements or, rather, may be due to the transmission of descending signals originating from primary motor cortex [57]. Another possibility is that cerebellar neurons fire before tic movements and their discharge takes place no later than that of primary motor cortex neurons [49].

Recently, McCairn and colleagues [49] have explicitly adopted a system-level approach to investigate the role of basal ganglia, cortical, and cerebellar areas in TS. The authors generated a pharmacologic motor tic/TS model with two monkeys by microinjecting the GABA antagonist bicuculline into the sensorimotor striatum (putamen) [57, 58]. In this way, the increased striatal inhibition caused abnormalities in the dopamine release [3, 59, 60] that, in turn, led to motor tics [13–16] (see section “Simulation settings” for more details). Neural activity was recorded from several areas of the basal ganglia, cerebellum, and primary motor cortex simultaneously to investigate their relationship. The results confirmed that aberrant activity leading to motor tics was initiated in the basal ganglia. However, they also showed how the occurrence of tics was closely associated with enhanced activity involving both the motor cortex and the cerebellum, implying that these may act in concert to produce overt tic movements. The time latencies of pathological activity in the cerebellum and primary motor cortex substantially overlapped and followed that of basal ganglia. This suggests that aberrant signals may travel along divergent pathways from the basal ganglia to the cortex and cerebellum. In this respect, the authors suggest that the basal ganglia might, presumably, influence cerebellar activity via the subthalamic-pons-cerebellar disynaptic link [30], with a latency that is sufficiently short to allow cerebellum to affect abnormal movements. However, the authors did not support this claim empirically.

Building on the results obtained in [49], in this paper we propose a computational model reproducing key anatomical and functional features of the system formed by the basal ganglia, thalamus, primary motor cortex, and cerebellum to investigate within a system-level perspective how motor tics are generated in TS.

The model yields several results and predictions. First, it reproduces the main results obtained in [49] about the differences in basal ganglia/primary motor cortex/cerebellum neural activity recorded during tic/no-tic events.

Second, and remarkably, the model shows that in order to reproduce and explain these data it is important to study the interplay between striatal dopamine signals and cortical activity, and the role played by the recently discovered subthalamic-pons-cerebellar pathway [30] working in synergy with the cerebello-thalamo-cortical circuit. In particular, the model predicts that the interplay between dopaminergic signals and cortical activity may underlie the emergence of tic events, and that the anatomical connection linking subthalamic nucleus and cerebellum may support the involvement of the cerebellum in tic production. In this way, the model supports the claim of [49] about a possible involvement of the subthalamic-pons-cerebellar circuit in tic generation, while specifying what functions it might accomplish. These predictions could form the basis for future experiments.

Third, the model predicts that tic production could be reduced by externally stimulating or inhibiting the primary motor cortex. These predictions could be important for identifying new target areas, aside the traditional ones [6, 61, 62], to design innovative system-level therapeutic actions.

**Fig 1:**
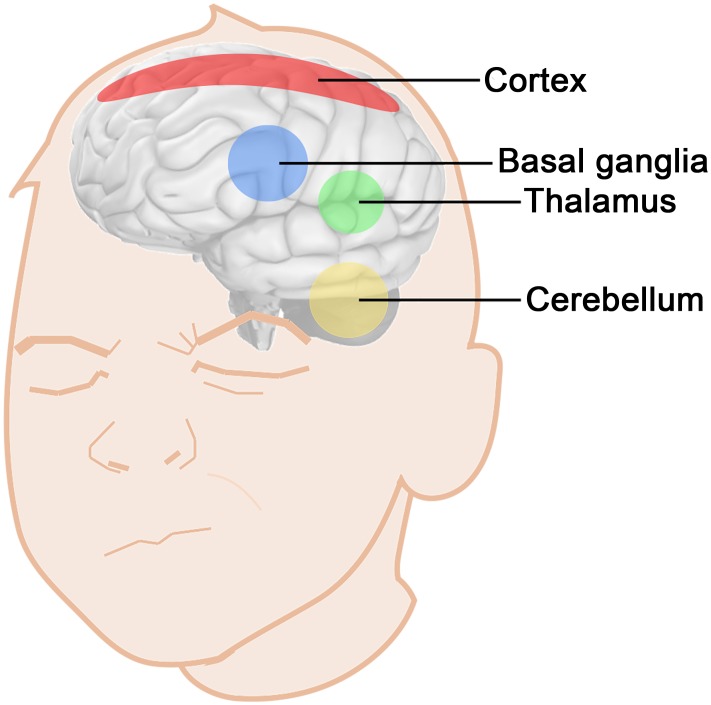
Key brain areas forming the “system” underlying tic production.

Finally, the model investigates the role of the recently discovered disynaptic bi-directional connections linking the basal ganglia with the cerebellum [29, 30]. To the best of our knowledge, there are no computational models investigating the role of these connections. Previous computational and conceptual models have, indeed, mainly studied the indirect interactions between basal ganglia and cerebellum mediated by cortical areas [63–69]. In view of recent empirical studies, attention to non-cortical-mediated basal ganglia-cerebellum interaction could radically change our perspective about how these subcortical areas interact with each other and with the cortex to regulate motor and non-motor behaviours [31, 35, 43, 55]. The computational model proposed here starts to address this issue by developing a simplified computational implementation of such links and by suggesting the possible involvement of the subthalamic-pons-cerebellar circuit in motor tic production. Fig 1 summarizes the brain areas mainly involved in tic production.

The rest of the paper is organized as follows. Section “Methods” describes the computational features of the model and the biological support of its assumptions. Section “Results” illustrates the results of the target empirical experiments with monkeys performed in [49] and how the model reproduces and explains them. It also presents the predictions of the model. Section “Discussion” discusses the system-level mechanisms through which the model explains the motor tic production and presents some limitations of the model while also suggesting possible future work to overcome them.

## Methods

### Architecture and functioning of the model

**Fig 2:**
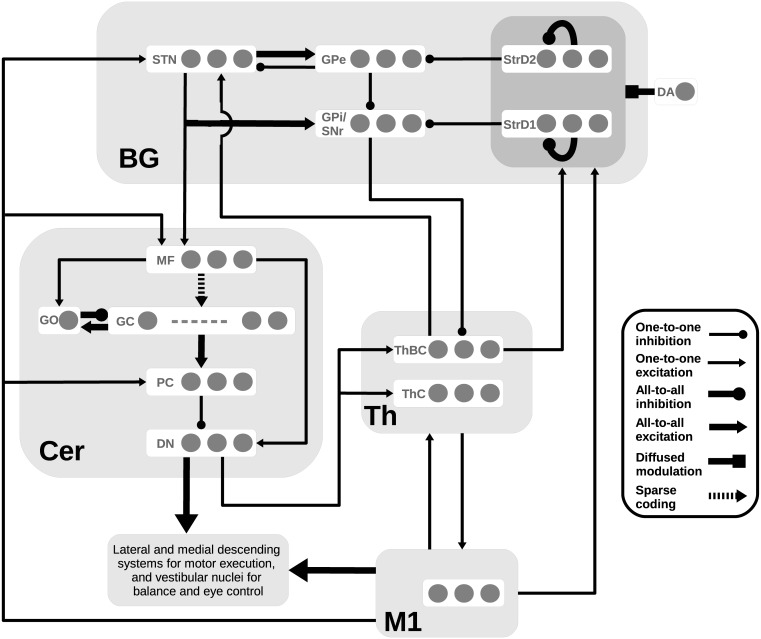
Architecture of the system-level model. The light gray boxes indicate the four components of the model: the basal ganglia component (**BG**), the cerebellum component (**Cer**), the thalamus component (**Th**) and the primary motor cortex component (**M1**). Each small dark gray circle within the components represents a leaky integrator unit whose activation potential represents the firing rate of a neural population. The three circles in each box represent three BG channels interacting with three different units of Th and M1. Both Cer and M1 project to the lateral and medial descending systems for motor execution, and vestibular nuclei for balance and eye control. Abbreviations: **StrD1**: D1 Receptor (D1R)-expressing striatal populations; **StrD2**: D2 Receptor (D2R)-expressing striatal populations, **STN**: subthalamic nucleus; **GPe**: external globus pallidus; **GPi**: internal globus pallidus; **SNr**: substantia nigra pars reticulata; **MF**: mossy fibers; **GC**: granule cells; **GO**: Golgi cell; **PC**: Purkinje cells; **DN**: dentate nuclei; **ThBC**: thalamic regions where both basal ganglia and cerebellum project; **ThC**: thalamic regions where only the cerebellum projects; **DA**: dopamine efflux regulated by a leaky unit and affecting both StrD1 and StrD2.

The system-level architecture of the model is formed by four main components (see Fig 2): the basal ganglia component (BG) reproduces the key anatomical and functional features of the basal ganglia building on the computational models proposed in [21, 70–72]; the cerebellum component (Cer) captures some critical anatomical and functional aspects of the cerebellum pivoting on the models proposed in [68, 73, 74]; the motor thalamus and the primary motor cortex components (respectively Th and M1), which do not focus on anatomical features, only reproduce functional aspects related to the activity of distinct neural populations. Indeed, as it was non-trivial to reproduce the dynamics of the complex system formed by the basal ganglia-thalamo-cortical loops, the loops linking the cerebellum with the cortex through thalamus, and the circuits linking the basal ganglia with the cerebellum, we used simplified models of the primary motor cortex and thalamus that allowed an easier study of the structures considered important for the generation of tics. This follows a strategy previously proposed for building system-level models more amenable to analysis [75] (cf. also [76, 77]). At the same time, due to the key role of the basal ganglia in triggering motor tics in TS [49] we considered a more sophisticated model of these nuclei with respect to the other components of the model. The possible effects of introducing finer grained anatomical and physiological details in the model are discussed in section “Conclusions and future work”.

With the exception of Cer components, each of the other model components is formed by three neural units representing three distinct neural populations encoding different information contents. From a behavioural point of view, it would have been sufficient to include just one neural unit for each component to address the target experiment of McCairn and colleagues [49]. Indeed, this experiment involved monkeys not solving any specific task but rather producing motor tics as spontaneous input-free behaviors under neural noise (as detailed below, in the model such noise is intended to capture the spurious effects on neural activation due to the signals supplied by other cortices as well as the effect of intrinsic neural noise [77–80]). However, it was important to include a larger number of neural units to reproduce in a realistic way the circuitry implementing the competitive dynamics typical of some components of the model, in particular of the BG [21, 81], relevant to the production of tics (see sections “The basal ganglia and their loops with the thalamo-cortical system: anatomy and physiology” and “The model predicts that the interplay between dopaminergic signal and cortical activity triggers the tic event”).

The neural units within each component of the model are represented by leaky integrator units [82, 83]. The activation of a single leaky unit represents the average firing rate of a population of real neurons. The neural population approach based on leaky integrator units is suitable for representing system-level features that are not immediately apparent at the level of individual neurons but manifest at higher levels [77]. This approach facilitates the comparison between the data on neural activation recorded in the model and the data obtained in the target experiment proposed in [49]. In addition, it allows a dimensionality reduction that increases the computational efficiency of simulations [84], and this is important for running sensitivity analyses of large models such as the one performed here. The chosen granularity of the model was also suitable for this work since it did not aim to reproduce detailed neural spatio-temporal patterns supporting the selection and performance of specific movements (cf. section “Simulation settings”).

The model has been implemented, as described here, based on a technique that was proposed in [85, 86] (see also [87]). This technique, suitable to illustrate neural system-level models formed by homogeneous neurons, aims to standardise all equations of the model so as to simplify its explanation, understanding, implementation, analysis, and reproducibility. The model is in particular fully described by the few equations presented in this section, the values of the equation parameters reported in the S2 Table (see Supporting Information), and the diagram of Fig 2 showing the architecture and connectivity of the model.

Each leaky integrator unit of the model components has an activation *a* and an activation potential (hereafter “potential”) *u* at time *t* having the following dynamics [82, 83]:

$$\tau \dot{u}=-u+I$$ (1)

a=f(u) (2)

where *τ* is the unit decay coefficient; *I* is the input to the unit that, depending on the component to which the unit belongs, could take into account the effects of the different pre-synaptic connections received from other components, the effects of noise, and the effects of dopamine. In particular, the term *I* of the post-synaptic unit *j* of the component *post* is computed as follows (the effects of dopamine are discussed below):

$$I_{post_{j}}=r_{post_{j}}+\sum_{pre}\sum_{i}w_{pre_{i}\to post_{j}}\cdot a_{pre_{i}}+n$$ (3)

where *r*_*post*_*j*__ is the resting potential of the post-synaptic unit *j* of the component *post*; *w*_*pre*_*i*_ → *post*_*j*__ is the weight of the connection from the pre-synaptic unit *i* of the component *pre* to the post-synaptic unit *j* of the component *post*; *a*_*pre*_*i*__ is the activity of the pre-synaptic unit *i* of the component *pre* computed according to Eq 2, and *n* is a noise value independently sampled from a Gaussian distribution for each unit. The pre-synaptic and post-synaptic units are those respectively sending and receiving signals as indicated in Fig 2. The function *f*(.) = [*tanh*(.) − *thr*]^+^ is the activation function of neural units, where *tanh*(.) is the hyperbolic tangent function, whose values were remapped to the range [−400, 400], *thr* is a parameter used to reproduce the effects of the threshold potential of real neurons [88], and [.]^+^ is a function returning the value of the function argument if this is positive, and zero otherwise. The differential equations related to the *u* of all units are numerically integrated using the Euler method.

### The basal ganglia and their loops with the thalamo-cortical system: Anatomy and physiology

Before presenting the computational details of the model components, this section highlights some features of the anatomy and physiology of the basal ganglia, and their loops with the thalamo-cortical system, as they are particularly important for tic production. The description uses the same abbreviations adopted for the model components shown in Fig 2.

#### The basal ganglia

The two main inputs stages of the BG are the striatum (Str) and STN. Str is formed by two subregions, StrD1 and StrD2. The StrD1 direct efferent projections originate from the medium spiny neurons and form the *direct pathway*. These projections are GABAergic and reach, through parallel channels, the internal globus pallidus (GPi) and the substantia nigra pars reticulata (SNr). The STN efferent projections represent the *hyper-direct pathway*. These projections are glutamatergic and spread diffusely over the external globus pallidus (GPe) and the GPi/SNr. Projections from StrD2 to GPe, and from there to GPi/SNr, represent the *indirect pathway*. These connections are GABAergic and organized in parallel channels similarly to those of the direct pathway [21, 24]. The direct pathway (StrD1) is formed by neurons expressing more D1-like affinity dopamine receptors, while the indirect pathway (StrD2) has neurons which tend to express more D2-like affinity dopamine receptors, even if recent data suggest a possible combined effect of both D1 and D2 receptors [89]. The direct pathway has a feed-forward organization while the indirect pathway is a recursive pathway which involves a negative feedback network. Indeed, the STN projections reach both GPe and GPi/SNr, with the difference that GPe also sends back inhibitory projections to STN [70, 90](see Fig 2).

#### The basal ganglia-thalamo-cortical loops

Most cortical areas (including M1) form re-entrant parallel loops with both BG and Cer [26, 35, 53]. In particular, the BG organization in parallel channels is also present in the circuit going from GPi and SNr to Th and then to M1 which projects back to StrD1, StrD2 and STN. Along this circuit, local populations have a relative segregation so that cortico-striato-nigro-thalamo-cortical parallel loops can be identified [24, 26, 27, 91]. There is wide evidence supporting the partial segregation of different loops involving the motor circuit while for the other higher-level cortical circuits, for example involving the pre-frontal cortex, the segregation is mainly inferred from comparisons of data on different components of each circuit obtained in distinct experiments [81, 92, 93].

#### The basal ganglia-thalamo-cortical loops for action selection

Various hypotheses have been proposed to explain how these loops are involved in movement processing [94, 95], or dimensionality reduction [96]. One influential perspective is that they have a key role in action selection [18, 19, 21, 22, 70, 90, 97]. In more detail, a GPi/SNr population of neurons reached by highly activated striatal afferents is inhibited while its neighbouring populations are excited by the STN glutamatergic projections. As a consequence, when the difference between the activity of two or more striatal areas is low, the difference in the inhibition of the activity of the corresponding SNr/GPi regions is high. This leads to a selective disinhibition of distinct thalamo-cortical loops. In addition, cortical feedback projections to StrD1, StrD2 and STN make the competition between channels a cumulative dynamical process. This process is similar of those described in neural-field modelling [82, 98, 99], with the difference that the competition within the cortico-striato-nigro-thalamo-cortical channels is based on disinhibition rather than excitation [100]. It has been proposed that the indirect pathway might control the activity passing through the direct and hyper-direct pathways [21], for example to normalise their activation with different numbers of activated channels. Other works suggest that action selection in the BG may rely on the existence of lateral inhibition among striatal spiny neurons [101]. This view is debatable since it has also been suggested that striatal spiny neurons are only weakly connected with insufficient lateral inhibition to completely support the action selection process [102]. In the computational model developed here, the action selection mechanism pivots on the interplay between the signals conveyed by the BG direct and indirect pathways in agreement with the hypotheses discussed in [21]. Importantly, the model predicts that the action selection mechanism helps explain the emergence of motor tics (see section “The model predicts that the interplay between dopaminergic signal and cortical activity triggers the tic event”).

### The Basal Ganglia component (BG)

In the model, the BG component includes five regions, each formed by a layer of three leaky integrator units. The two main inputs of the BG component are Str and STN. Str is formed by two subregions, StrD1 and StrD2, with units expressing D1R and D2R dopamine receptors. STN works in a loop with GPe and receives most of its afferent projections from M1. Similarly, StrD1 and StrD2 receive afferent projections from M1 and Th. StrD1, StrD2, STN and GPe send efferent projections to the GPi or SNr, which are the GABAergic output nuclei of the BG (hereafter, GPi and SNr, represented as one component in the model, will be indicated as GPi/SNr).

The excitatory and inhibitory connections between the regions of the BG component are feedforward links between one unit and the topologically corresponding unit in the following layer (thin lines in Fig 2). This connectivity reproduces in an abstract fashion the structure of the BG channels (one-to-one connections). The units of STN are connected with all GPi and GPe units (all-to-all connections). This simulates the diffused action of the STN over its target regions [24, 70]. BG project to the Th through inhibitory links (GPi/SNr-Th) [21] and to Cer through excitatory connections (STN-Cer) [30, 35].

For the striatal sub-component StrD1, the *I* term is calculated by multiplying the right side of Eq 3 for the dopaminergic term *a*_*DA*_*D*1__ used to account for the dopaminergic modulation on the activity of StrD1 and computed as follows:

$$a_{DA_{D1}}=b_{StrD1}+d_{StrD1}\cdot a_{DA}$$ (4)

where *b*_*StrD*1_ is a baseline StrD1 potential modulation not due to DA, *d*_*StrD*1_ is the StrD1 DA factor amplitude, and *a*_*DA*_ is the activity of a leaky integrator unit (Eq 2) used to simulate the dopamine efflux. The dopamine efflux was simulated through an activation potential *u*_*DA*_ of the DA leaky unit that rapidly reaches a maximum level *DA*_*MAX*_ = 0.5 around 1 *sec* from the beginning of each trial, and then decays toward *DA*_*MIN*_ = 0.01.

Similarly, for the striatal sub-component StrD2 the *I* term is calculated by multiplying the right side of Eq 3 by the dopaminergic term *a*_*DA*_*D*2__ used to account for the dopaminergic modulation on the activity of StrD2 and computed as follows:

$$a_{DA_{D2}}=\frac{a_{DA_{D1}}}{b_{StrD2}+d_{StrD2}\cdot a_{DA}}$$ (5)

where *b*_*StrD*2_ is a baseline StrD2 potential modulation not due to DA and *d*_*StrD*2_ is the StrD2 DA factor amplitude.

While the contribution of the dopaminergic efflux on the activity of StrD1 units was implemented as a *multiplicative excitatory* effect (Eq 4), the modulation of dopaminergic efflux on the activity of StrD2 units was implemented as a *multiplicative inhibitory* effect (Eq 5). It has been shown that these two different types of dopaminergic modulations reflected what happens in the real BG (cf. [103]). Hence the term *a*_*DA*_*D*1__ in the Eq 5 takes into account the recent data showing a possible combined effect of D1 and D2 receptors [89]. For the other sub-components of BG (STN, GPe and GPi) the *I* term was computed by simply using the Eq 3.

### The Thalamus component (Th)

The Th component is formed by two regions: ThBC, representing the thalamic parts where both BG and Cer project; ThC, representing the thalamic areas where only Cer projects. Each region includes three leaky integrator units. This organization in two subregions is based on anatomical data showing the presence of both partially segregated and overlapping projections from the BG and Cer output regions to Th [104, 105]. ThBC receives inhibitory signals from the BG component (GPi/SNr region) and excitatory signals from the Cer component [106, 107]. By contrast, ThC only receives excitatory signals from the Cer component [26, 105]. In addition, ThBC and ThC send excitatory signals to the input stages of the BG component (StrD1, StrD2, STN) [29, 55, 108, 109] and are bi-directionally connected with M1 through excitatory links [26, 27, 53]. The *I* terms of ThBC and ThC were computed using Eq 3.

### The Cerebellum component (Cer)

The Cer component was built starting from the Marr-Albus type of model [110, 111] proposed in [68, 73, 74], as these are implemented with a level of abstraction that was similar to the one of the BG component. In particular, the Cer includes four regions, each formed by a layer of leaky integrator units: the granule cells (GC) formed by 100 units; the Golgi cells (GO) formed by one inhibitory unit; the Purkinje cells (PC) formed by three units; the dentate nuclei (DN) formed by three units. These numbers approximate the proportion of neurons observed in the real Cer [110–112]. There is also a mossy fibers (MF) layer which receives excitatory connections from M1 and STN. These circuits reproduce the functional effects of the M1 and STN activities on the cerebellar areas due to the pons-cerebellar link [26, 30]. GC transform the signal from MF for further processing by the PC. According to the Marr-Albus theory, GC provide a sparse code, that is, a code with only a small fraction (less than 10% in the model used here) of cells active at any time. In this way, the functioning of the cerebellum is facilitated because different MF inputs create highly dissimilar sparse GC activity patterns, which are easily recognizable by PC. GO receives excitatory input from MF and GC, and provides a feedback inhibition to GC. GO firing suppresses MF excitation of GC and thus tends to shorten the duration of bursts in the connections linking GC to PC. This mechanism further supports the sparse coding of the input [73]. PC show a spontaneous activity [112] that is influenced by parallel fibers—these are excitatory afferent inputs from GC.

PC also receive an input signal from M1 through the inferior olive-climbing fiber system—a climbing fiber is an axon of a neuron of the inferior olive. This circuit is important for implementing Cer learning processes [113]. In this respect, the inferior olive is commonly thought to compute an error signal conveyed to PC through nucleo-olivary projections (refer to [114] for a detailed computational model). In particular, in a model that would take into account the Cer learning processes, the output of DN should be subtracted from the M1 input to PC. The inferior olive-climbing fiber system is also relevant to managing the timing of the input [115]. Since the model did not aim to study the effects of Cer learning processes on tics, we abstracted the timing effect of such a system with a simple connection from M1 to PC (see Fig 2). This link contributes to modulate the PC activity in a synchronous way with respect to the M1 activity. The activity of the units of DN is modulated by the inhibitory connections from the corresponding units of PC (one-to-one connections) and by the excitatory collaterals from MF supplying a baseline activation for DN [116]. The three units of DN, in turn, send excitatory signals to M1 (through Th) [26] and to StrD1 and StrD2 (through ThC) [29, 105].

The basic functioning of the Cer component is organized around the inhibitory PC, whose axons provide the only output of the cerebellar cortex. Each unit of PC modulates the selection of a particular motor pattern within the dentate-thalamo-cortical system [117]. In other words, similarly to what happens to BG, parallel sub-loops with the Cer component independently modulate a motor pattern allowing the selective facilitation of one response and the concurrent suppression of the others [26, 35, 45]. When MF are silent (i.e., no input is received by Cer), PC show spontaneous activity and their inhibitory output prevents DN cells from firing. This in turn prevents the selection of responses at such times. We assumed that a previous learning process based on long-term depression (LTD) and long-term potentiation (LTP) [68, 118] has led to having the GC-PC connections assume a high negative value when a motor pattern has to be selected by the input, and a small negative or positive value when a motor pattern should be inhibited. The high negative value for the GC-PC synapse assures that the activity of the corresponding PC unit is close to zero and this in turn makes the corresponding DN unit positively activated [111]. Consequently, excitation from MF collaterals predominates over inhibition from PC to DN related to the correct response. DN neurons excite the thalamus that, in turn, excites the region in the motor cortex related to the correct response.

The *I* terms for the units of the Cer were computed using Eq 3. The noise term *n* was set to zero for GC, GO, PC and DN. We set by hand the value of the elements of *w*_*GC* → *PC*_ by assuming that a previous learning process had led activity from GC to PC having a zero value when a motor pattern has to be selected by the input, and a positive value when no motor pattern has to be selected. The values of the parameters of the equations are shown in the S2 Table (see Supporting Information). The activation recorded in the GC and PC layers is assumed to correspond to the firing rates measured within the cerebellar cortex of the monkeys (labeled as “CbllCx” in [49]).

### The primary motor cortex component (M1)

The M1 component is formed by three leaky units whose activity is assumed to correspond to the firing rate recorded in the primary motor cortex of the monkeys in the target experiment of McCairn and colleagues [49]. M1 is bi-directionally connected with Th and projects to BG and Cer through excitatory links [26]. The *I* term of the units of the M1 component was computed using Eq 3.

### Simulation settings

#### Trials

The equations of the model were numerically integrated using the Euler method with an integration step Δ*t* = 0.001 (i.e., one simulation step corresponded to 0.001 *sec* of real time). The simulation was run for 90 *sec*. Within this time, we monitored the neural activity of the model components in 10 time windows (“trials”) each lasting 2 *sec* (2000 simulation steps) and separated by a 7 *sec* time interval to avoid the neural activity of each trial influencing the subsequent trial. The trials in the simulation corresponded to the time intervals studied in the target experiment [49]. Within each of these trials a tic could possibly occur as a consequence of the dopamine manipulation. In the model, within each trial, after 1 *sec* from its beginning, a dopamine efflux was produced by activating the dopaminergic unit to simulate the effects of bicuculline injection. The dopamine first reached a peak (*DA*_*MAX*_) and then gradually decayed towards a low value (*DA*_*MIN*_).

#### Intervals in which a tic does or does not occur

In the model, we distinguished the trials with and without tics to form two classes and datasets called respectively “TIC” and “NO-TIC”. To classify the trials into one or the other class, in each trial we computed the time average activity shown by each of the M1 units: M1 was indeed expected to exhibit a substantial activity difference when a tic was produced compared to when it was not produced. If the average activity peak amplitude in M1 was above a threshold of 40, the trial was considered to be part of the TIC class, otherwise the trial was considered to be part of the NO-TIC class. We verified through preliminary tests that the threshold value of 40 removed the trials where the average activity peak amplitude of M1 units was small. In NO-TIC trials, the relative values of the noise signals of the units within the direct/indirect pathways stages did not allow the starting of the action selection process leading to produce a tic (cf., section “The model predicts that the interplay between dopaminergic signal and cortical activity triggers the tic event”).

The activity of M1 during motor tics was not used to produce overt movements through, for example, a “reading out” procedure controlling an embodied system such as a real/simulated humanoid robotic arm and hand (cf., [119–122]). This was indeed unnecessary as we were interested in reproducing only the data from [49] on neural activation and not data on kinematic and dynamic aspects of tics such as those presented in [123].

#### Repetitions of the experiment

Wheras the target data were collected in two monkeys, we collected data from 40 different simulated subjects (10 trials per subject). The different subjects were obtained by running the model with different seeds of the random number generator which in turn caused different values of the noise signals. In this way, we exploited the ability given by the simulation approach to produce many subjects with a minor cost so as to have a higher statistical reliability of the results.

#### Conditions to have motor tics

The authors of [49] microinjected the GABA antagonist bicuculline into the sensorimotor striatum (putamen) of two monkeys to generate the conditions potentially producing tics [57, 58]. The bicuculline causes an increase of the striatal inhibitory activity through a local disinhibition due to the blockade of GABA. It has been shown that the increase of striatal inhibition causes abnormalities in the tonic/phasic dopaminergic releases [3, 59, 60] which in turn can lead to motor tics [13–16].

For simplicity, in the computational model we directly increased the striatal dopamine efflux in order to simulate the effect of the increased striatal inhibition caused by the bicuculline injection. In this way, we could also study the effects of different levels of dopamine on tic production. The increase in the dopamine efflux was simulated by the activation potential of a leaky unit representing a population of dopamine neurons that fired after 1 *sec* from the beginning of the trial and then decayed toward a low value. This pattern of dopamine release agrees with data showing that dopamine concentrations in the striatum of rats change during significant behavioral and pharmacological events, such as copulation and administration of drugs [124, 125].

As shown in the next section, the increased striatal inhibition in the experiment and the abnormal burst of dopamine in the model represent necessary but not sufficient conditions to have motor tics. Indeed, the production of tics also needs a concomitant activation of cortex, possibly selected and amplified by BG.

#### Tuning the parameters of the model

S2 Table (see Supporting Information) shows the values of the parameters of the model. The values not marked with a star were heuristically set starting from the values they assumed in the original models of the BG [21, 72] and cerebellum [73] from which we started to build the current model. Some of these parameters are critical for the basic functioning of the inner circuits of BG and Cer related to the selection of motor patterns based on the interplay of the BG direct and indirect pathways and the Cer PC inhibition. The values of the original models were justified in [21, 72, 73] on the basis of physiological considerations.

The setting of the remaining parameters (those marked with a star in S2 Table) was done through an automatic optimization procedure, namely a “genetic algorithm” [126] (see [103, 127] for similar approaches using genetic algorithms). The algorithm went through optimization cycles called “generations”. The parameter search started from randomly generated candidate solutions. For each generation, a “population” of candidate solutions corresponding to different possible sets of model parameters was “evolved” to minimize the mean square error between the firing rate of the model units and the neural activity drawn from the target experiment [49]. The error was normalized in the range (0, 1). A low value of such error indicated that the activity exhibited by the model was similar to the data collected from the real subjects. We heuristically set the range of possible values that each parameter could assume by relying on the values they assumed in the models of basal ganglia-thalamo-cortical loops and dopaminergic system we developed in the past [71, 103, 128, 129] as well as on the basis of physiological considerations drawn from [21, 72, 73, 90].

The search explored 2500 candidate parameter sets, and hence models (during 300 generations of the genetic algorithm), to arrive to an error smaller than 0.08, a value that guaranteed a good matching between the simulated and the real data. The optimisation was run through the computers of the *Neuroscience Gateway (NSG)* portal [130] and the *Grid’5000* system [131], both allowing free access and use of high performance computing resources. The parameters marked with a star in the S2 Table are the best ones obtained with this procedure. Importantly, alongside the parameters giving the lowest error we also saved all the other parameter sets explored by the algorithm and causing a higher error. These data were used to run a sensitivity analysis of the model behaviour with different values of the parameters, as illustrated in section “Results”. The model was developed using the Python programming language. The code of the model is available from this link https://github.com/locen/Tourette-model.

## Results

This section presents the results of the simulations run with the model and directed to: (i) *reproduce recent data* from [49] on firing rates during tics/no-tic events recorded in several areas of the system formed by BG, M1, and Cer; (ii) *understand* the system-level mechanisms underlying such phenomena; (iii) *produce predictions* on tic-related abnormal activities in regions not investigated in the experiment with real subjects (e.g., STN and Th), on how the tic generation changes with an increase of striatal dopamine, and on how it changes with a modulation of the activity of M1.

The target data addressed by the model were drawn from the work of McCairn and colleagues [49]. Note how in this respect the model architecture and functioning are empirically supported by two kinds of constraints: the “general” constraints on anatomy and physiology of the basal ganglia-cerebello-thalamo-cortical system (see section “Methods”); and the “specific” constraints consisting in the fact that the model was required to reproduce the target data obtained in [49]. The model could satisfy both sets of constraints only after its parameters were suitable tuned. This multi-level empirical validation of the model increases the plausibility of the explanations of the system-level mechanisms underlying the target data (point (ii)) and of the predictions made by the model (point (iii)).

### The model reproduces data on firing rate during tic/intertic intervals

**Fig 3:**
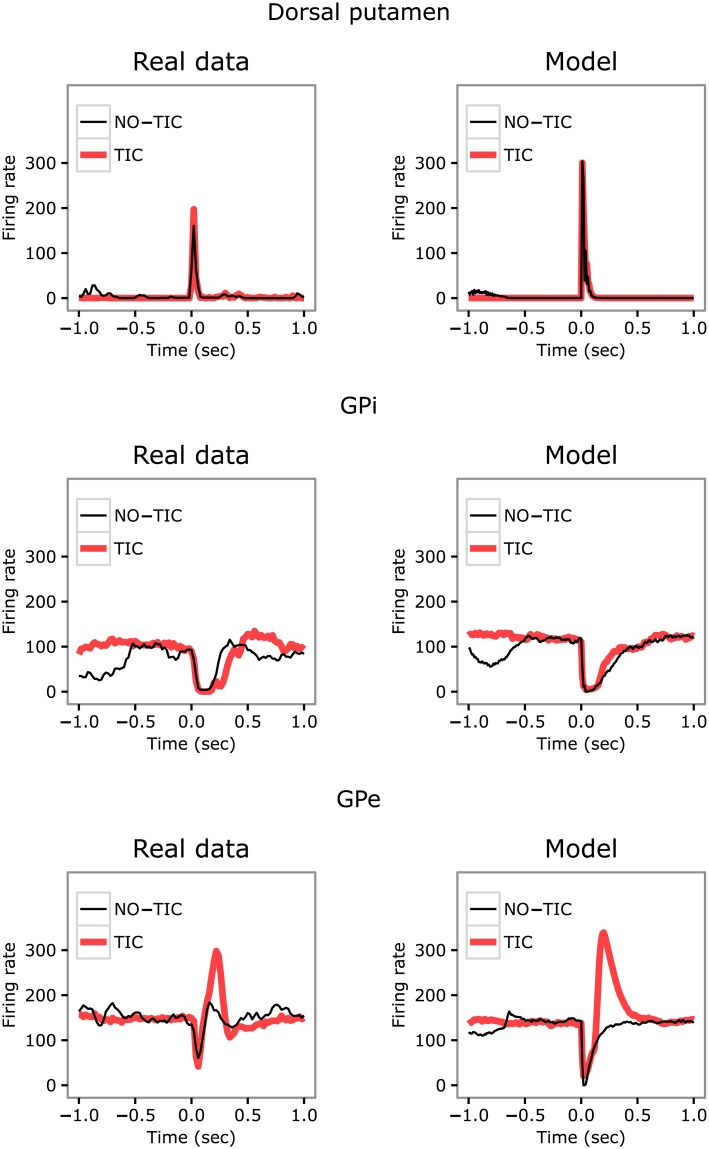
Firing rate within the basal ganglia during tic and intertic time intervals. Left: data recorded in the monkey. Right: same data recorded in the model. First row: Dorsal putamen activity in the intertic (thin-black line) and tic (thick-red line) intervals. Second row: activity from GPi in the two intervals. Third row: same data for the GPe. The real data were extracted from figure 8 of [49] (reprinted with permission).

**Fig 4:**
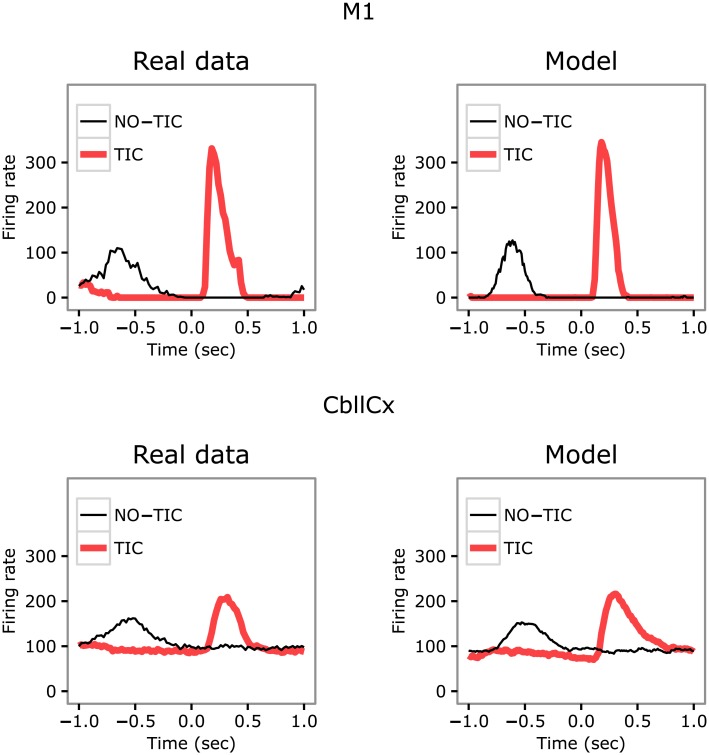
Firing rate in the primary motor cortex and cerebellum during tic and intertic time intervals. Left: data recorded in the monkey. Right: same data recorded in the model. First row: differential M1 activity between the intertic (thin-black line) and tic (thick-red line) intervals. Second row: same data for CbllCx. The real data were extracted from figure 8 of [49] (reprinted with permission).

This section compares the data on neural activity collected in [49] in the brain of one monkey and the data on neural activity collected in the brain of one subject simulated with the model (data for other subjects are qualitatively similar). Figs 3 and 4 show respectively the firing rate in the BG and in the M1 and Cer during TIC and NO-TIC trials (i.e., intertic intervals) recorded in the monkey and in the model.

The model curves are obtained with (a) an activation of cortex affected by the intrinsic neural noise of the various regions of the model; (b) a further activation mimicking possible inputs to M1 from other cortical areas (here captured, in the case of no-tic and tic cases, with a Gaussian-like input with a height of respectively 30 and 17, and a standard deviation of respectively 0.040 and 0.250 *sec*); (c) dopaminergic bursts that capture the possible dopamine dysregulation caused by bicuculline (here captured, in the case of no-tic and tic cases, with a Gaussian-like input with a height of respectively 1 and 50 and a standard deviation of respectively 0.600 and 0.020 *sec*).

The figures show that real and simulated data are very similar. In both cases, in the Dorsal putamen and GPi there are no relevant differences in the firing rate amplitudes between the tic and no-tic state whereas there is a partial preservation of the response for GPe, with the early inhibitory peak maintained and the later excitatory peak increased during a tic. By contrast, for M1 and Cer (CbllCx in the figure) the firing rate amplitudes during the tic state are greater than those measured during the no-tic state.

### The model predicts an abnormal tic-related activity in the subthalamic nucleus and in the thalamus

**Fig 5:**
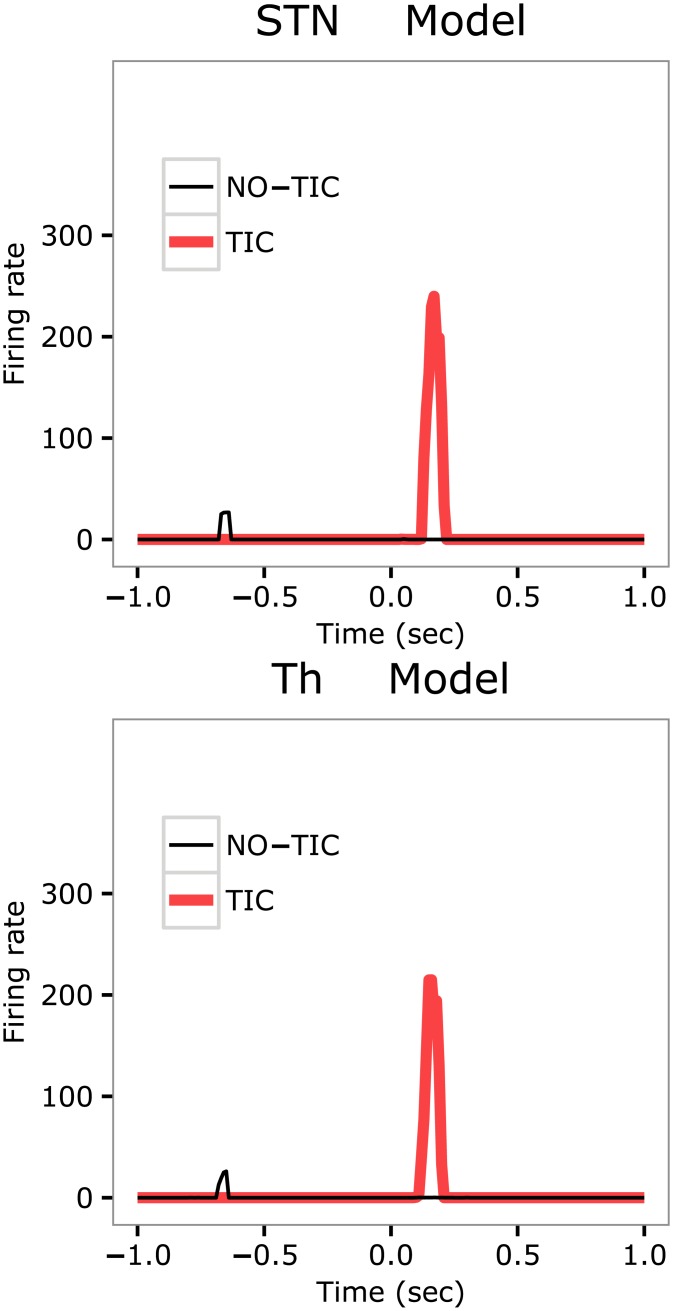
Activity of the model subthalamic nucleus and thalamus in TIC and NO-TIC trials. Top: Subthalamic nucleus. Bottom: Thalamus.

The model allows the simulation of the activity of other key areas not monitored in the target experiment [49]. In particular, we measured the activity in STN and Th based on the hypothesis that these regions might be involved in tic production due to their potential role as mediators between M1, BG, and Cer signals [26, 55, 105]. Fig 5 shows that, similarly to what happens for M1 and CbllCx, in the STN and Th there is a remarkable difference in the activity amplitudes between tic and no-tic states. This result represents a prediction of the model that could be tested in new experiments. The abnormal activation of M1 in case of a tic supports the increase of activity in STN and Th. The enhanced activity of STN, in turn, contributes to get a larger excitatory peak in GPe (cf. section “Propagation of aberrant basal ganglia activity to primary motor cortex and cerebellum”). In section “Discussion”, we further discuss the possible neural processes based on which STN and Th may be involved in tic production.

### Statistical analysis

**Fig 6:**
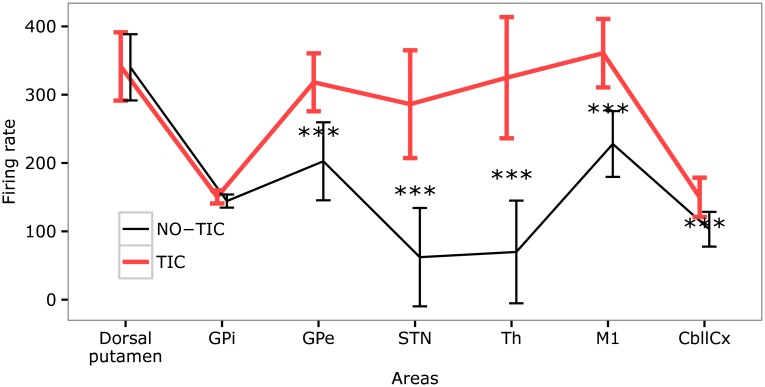
Statistical comparison between average value and standard deviation of the peak amplitude of the activity in different areas of the model, involving 40 simulated subjects. The black-thin line indicates the values computed in no-tic events (NO-TIC); the thick-red line refers to the values computed in the tic events (TIC). Statistically significant differences are indicated with three stars.

The results obtained with the model and presented in sections “The model reproduces data on firing rate during tic/intertic intervals” and “The model predicts an abnormal tic-related activity in the subthalamic nucleus and in the thalamus” are supported by statistical analysis of the data collected across 40 simulated subjects. For each subject, we considered one trial randomly selected from the 10 trials. In this way, we got 40 different measures across all the simulated subjects. A two-way analysis of variance (ANOVA) was performed using the function *aov* of the statistical analysis software *R*. In more detail, the ANOVA was performed with two factors, namely the peak activity in the different areas (i.e., Dorsal putamen, GPi, GPe, STN, Th, M1, CbllCx) and the movement state (i.e., NO-TIC vs. TIC). A post hoc test was also applied using the function *TukeyHSD* of *R*. A result was considered statistically significant if the *p* value was less than 0.001. The average value of the peak amplitude of the activity and its standard deviation for the areas of the model in TIC and NO-TIC trials are reported in the S3 Table, visually summarised in Fig 6. The ANOVA shows a statistically significant interaction between the activity in the different areas and the movement state (*p* < 0.001). In addition, the post hoc tests show that, as in the experiment of McCairn and colleagues [49], the differences in the activity amplitudes between TIC and NO-TIC trials are not statistical significant for the Dorsal putamen (*p* = 0.990) and GPi (*p* = 0.970), whereas they are statistically significant for all other regions, in particular GPe, STN, Th, M1, and CbllCx (*p* < 0.001 for all of them).

### Propagation of aberrant basal ganglia activity to primary motor cortex and cerebellum

**Fig 7:**
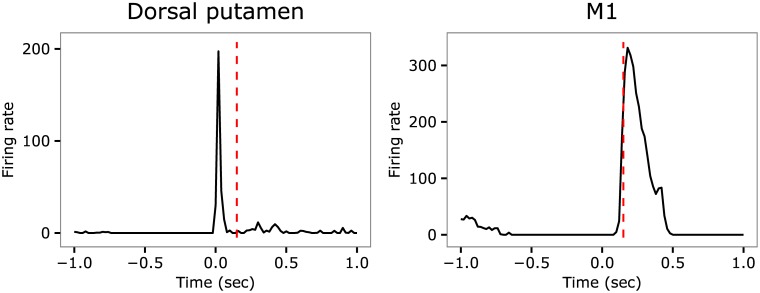
Firing rate in the Dorsal putamen (left) and in the primary motor cortex (right) recorded in the real experiment by [49]. The dashed-red vertical line indicates the time of tic onset. Data adapted from figure 8 of [49] (reproduced with permission).

Fig 7 shows the firing rate of the Dorsal putamen and M1 cells presented in [49] and obtained by recording neuron activity in the monkey model of tics. The authors found that the striatal burst occurs 0.29 *sec* before the tic initiation. This is followed by the activation of GPe and GPi, occurring respectively 0.26 *sec* and 0.19 *sec* before the tic onset, and by the activation of Cer and M1 respectively happening 0.11 *sec* and 0.12 *sec* before the tic onset. The authors also found significant differences in the latency distribution of BG areas versus M1 and CbllCx, whereas they did not find significant differences in this distribution between M1 and CbllCx. Overall, these findings suggest that in the animal model of [49] the tic event is triggered by the putamen as the activation of BG precedes that of M1 and Cer.

**Fig 8:**
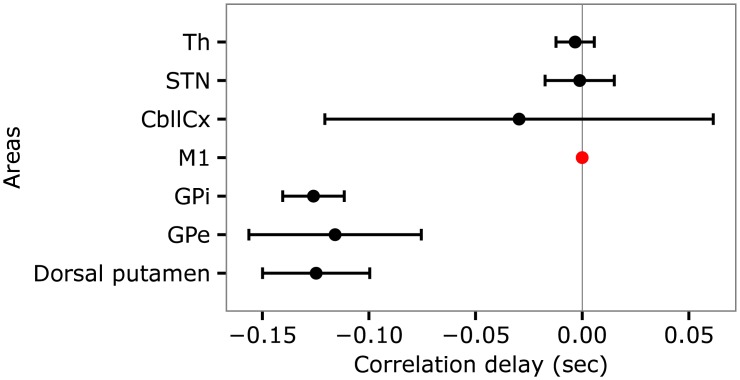
Delays between the onset of the average activity in M1 and the onset of the average activities in the other areas of the model in the case of a tic. The black dots indicate the means whereas the bars indicate the standard deviations. The red dot indicates the reference with respect to which the cross-correlation was computed.

We obtained similar results in the model. In more detail, to study the causality of the signal propagation in the model we computed the delay of the onset of the average activity in M1 with respect to the onset of the average activities in the other areas. The delay was calculated by using the cross-correlation function *ccf* of the statistical analysis software *R* applied to the derivative of the signals. The results of the cross-correlations are summarized in Fig 8. The figure shows that in the tic state the onset of the average activity in the Dorsal putamen takes place 0.126 *sec* before the onset of the same signal in M1. Similarly, the onset of the average activity in GPe and GPi anticipates the onset of the same signal in M1 of respectively 0.116 *sec* and 0.124 *sec*. By contrast, the delays between the onset of the average activity in STN, Th, CbllCx and in M1 are small.

We performed a statistical analysis over the data collected in 40 simulated subjects (the data for the analysis were collected as described in section “Statistical analysis”). The one-way ANOVA (having as a factor the means of the delays for each area) shows that there are significant differences in the means of the delays resulting from the cross-correlations between M1 and the other areas (*p* < 0.001). The post hoc tests show that there are statistically significant differences between the means of the delays related to Dorsal putamen, GPi, and GPe and the mean related to M1 (*p* < 0.001 for all comparisons). By contrast, there are no statistically significant differences between the means of the delays related to STN, Th, and CbllCx and the mean related to M1 (M1 vs. STN: *p* = 0.988; M1 vs. Th: *p* = 0.991; M1 vs. CbllCx: *p* = 0.156). The clustering in two groups of the delays is apparent from Fig 8. Overall, these results suggest that in the model the abnormal tic-related activity in M1 is triggered in the Dorsal putamen and propagates through GPe, GPi, STN, Th, and CbllCx.

### The model predicts that the interplay between dopaminergic signal and cortical activity triggers the tic event

The data shown in the previous section suggest that the tic activity first emerges in the BG, in particular in the Dorsal putamen, and then propagates towards the other regions of BG, and to Th, Cer, and M1. However, these data do not answer the question: why in some trials is there a tic event while in others there is not? The model suggests a possible answer to this question. In more detail, the model predicts that in the case of trials where a tic is exhibited there is a conjunction of two events: (i) a dopaminergic burst; (ii) M1 neurons activation happening at a time close to the dopaminergic burst and due to noise and inputs from other cortical areas.

**Fig 9:**
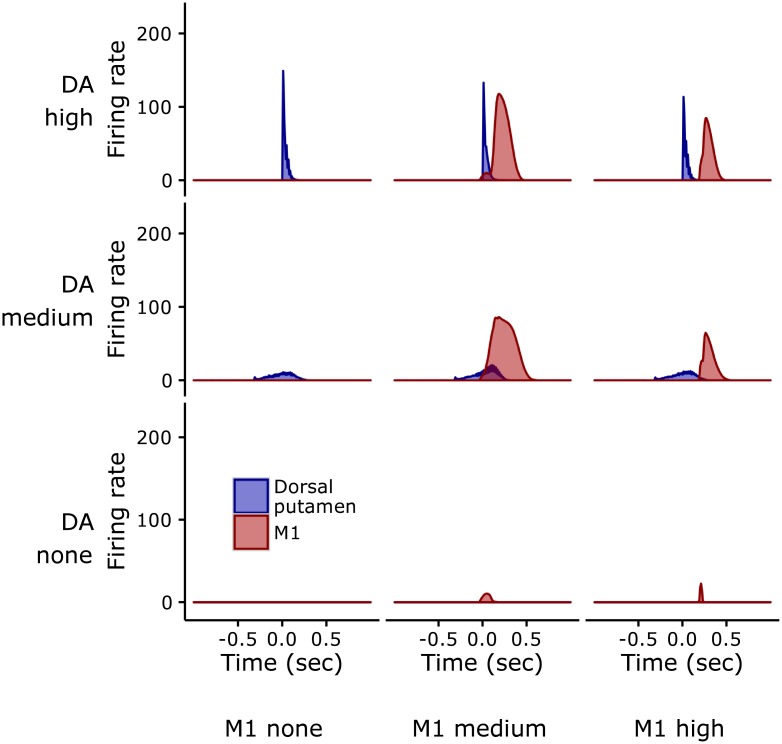
Effects of the interactions between dopamine bursts and M1 activations on tic/no-tic production. Each graph represents the firing rate of Dorsal putamen (blue trace) and M1 (red trace) for different combinations of three possible levels of dopamine bursts and M1 activations. The three activations of M1 had a shape as the one shown for the DA-none condition.

To further investigate this mechanism of tic generation, we ran further simulations where we explicitly simulated different random events possibly affecting dopamine, representing the effects of dopamine dysregulation caused by bicuculline, and M1, possibly representing inputs from other cortical regions. The interaction of cortical and dopamine events having different intensities are shown in Fig 9.

The graphs of the figure have been obtained with three increasing levels of M1 activation (simulated with a Gaussian-like input with a height measuring respectively 0, 17, and 30, and a standard deviation measuring respectively 0.250 and 0.040 *sec* for the two non-zero height cases). Such input was also multiplied by a random number drawn from a uniform distribution ranging in (0, 1) before being sent to each of the three channels of M1, so as to capture differential inputs received by the three channels. For dopamine, we simulated three dopaminergic bursts with increasing intensities (simulated with a Gaussian-like input with a height measuring respectively 0, 1, and 50, and a standard deviation measuring respectively 0.600 and 0.020 *sec* for the two non-zero height cases).

**Fig 10:**
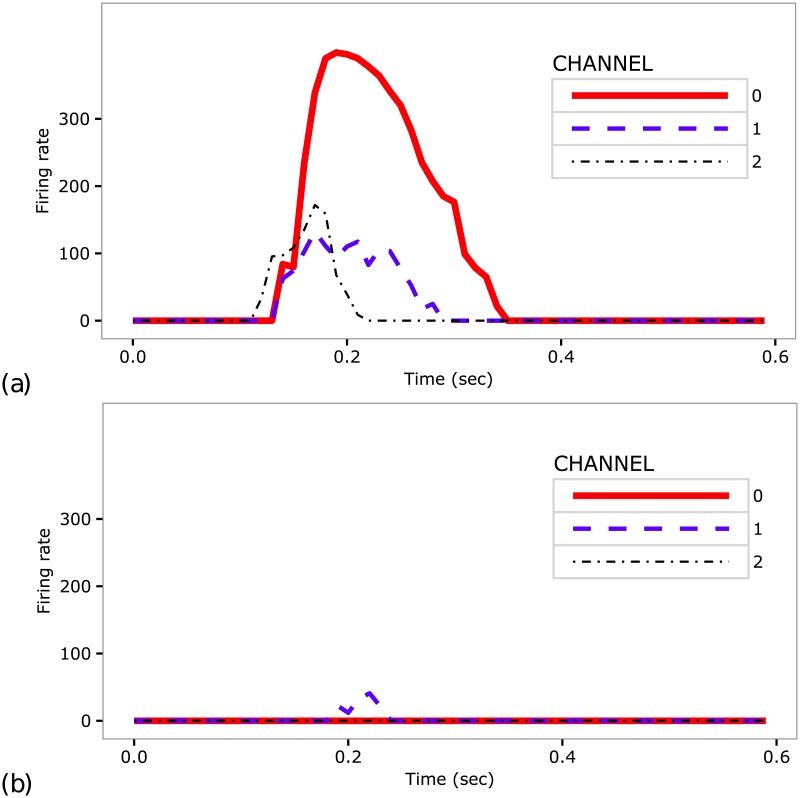
Activity of the three units of primary motor cortex. Data recorded in the case of (a) tic and (b) no-tic. In the case of a tic, the noise signal conveyed by one channel wins the competition and causes a strong activation of the related cortical unit, whereas all cortical units remain silent in the case of no-tic.

The figure shows that when the two events occur together and have a sufficient intensity, the activity of the Dorsal putamen triggers the BG selection process (see section “The basal ganglia component (BG)”). In this way, the noisy signal conveyed to one of the three channels is possibly amplified so that it wins the neural competition. The signal of the winner channel is then transmitted to the Cer through the STN-pons-Cer circuit and further modulated through the Cer-Th-M1 circuit, contributing to an abnormal activity in STN, Cer and M1. The activity within M1 is also amplified through the recurrent excitatory M1-Th loop. Fig 10a shows the effects on the activation of the three M1 channels and tic production caused by the concomitant occurrence of M1 activation and a dopamine burst.

By contrast, the model does not exhibit tics if there is no relevant activity of M1 (Fig 9). Indeed, in this case even if the Dorsal putamen might have an activity due to the striatal noise and the dopamine production, the BG selection process cannot select any signal within the BG-Th-M1 channels as the thalamic activity is substantially zero. Similarly, the model does not exhibit tics if there is a non-zero activity of M1 but there is not a dopaminergic burst. The reason is that the activity of Dorsal putamen depends on the presence of dopamine. Dopamine modulates the signals conveyed by the direct and indirect pathways in different ways: it has a multiplicative effect on StrD1 (cf. Eq 4) and an inhibitory effect on StrD2 (cf. Eq 5). If the dopamine burst is zero (or close to zero) the Dorsal putamen shows a very low activity (Fig 9) and cannot support the selection process. In particular, the signal transmitted by the indirect pathway leads to a strong net inhibitory effect on the signals conveyed by the three BG-Th-M1 channels. This implies that no channel can win the neural competition and so no tics are generated (Fig 10b).

As mentioned, Fig 9 has been obtained by directly activating M1 units through an external signal. This simulated process might be thought to mimic a real situation where cortex is activated through an external stimulation. As an example, this stimulation might be performed through *transcranial direct-current stimulation* (tDCS), which can be used to either enhance or inhibit cortical activity. The model hence suggests the possibility of designing tDCS non-invasive treatments targeting M1 and directed to induce suitable plasticity processes possibly reducing tic generation [132].

### The model predicts that the number of tics increases with dopamine

**Fig 11:**
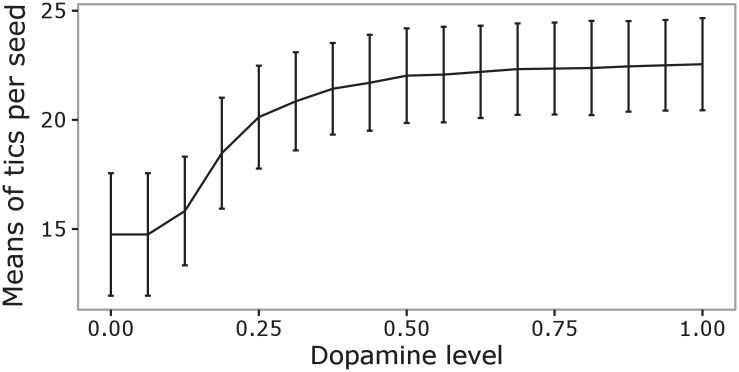
Number of tics with different levels of dopamine. The dots indicate the dopamine level averaged over 40 simulated subjects in correspondence to different levels of dopamine, the vertical lines indicate the standard deviation.

We further investigated the role of dopamine in tic generation by running simulations where we gradually increased the level of the dopamine bursts. In particular, we considered dopaminergic bursts having a peak that increased from 0 to 100 in 17 steps (the burst had a Gaussian shape with 0.020 *sec* of standard deviation). M1 received random inputs simulating afferent signals received from other cortical areas. The inputs had a Gaussian-shape in correspondence to the dopamine bursts, in particular they had a height randomly drawn from the range of (0, 900) and had a standard deviation of 0.040 *sec*. For each level of dopamine, we analysed the data collected in 30 trials of 40 simulated subjects. The results are shown in Fig 11. The model predicts that the number of tics progressively increases with the size of the dopamine bursts. This result confirms what was said in relation to Fig 9, showing how stronger dopaminergic bursts lead to a higher probability of producing tics.

A statistical analysis supports the results shown in Fig 11. In particular, the data were analysed through a one-way ANOVA having as factor the dopamine levels. The ANOVA shows that the dopamine level has a significant effect on the number of tics (*p* < 0.001).

### Sensitivity analysis: Effects of the model parameters on the model predictions

We evaluated how the data collected with the model were sensitive to the variations of the values assumed by the parameters when running the optimisation procedure discussed in section “Simulation settings”. In particular, we restricted the analysis to the best parameter sets found by the optimisation procedure during the whole search, namely to those that produced a high fit of the model to the target empirical data. To this purpose, we selected the parameter sets having a fitting data error within the first quartile (this amounted to selecting the parameter sets having a simulated-real data error smaller than 0.08). The analyses focused on the standard deviation of the (normalized) values of the parameters sets selected in such a way. The focus on the standard deviation was based on the idea that a small variance of a parameter indicated a great influence of the parameter on the model behaviour: indeed, the values of the parameter which were far away from its mean were associated, with a high probability, with a worse data fitting by the model and so were discarded by the procedure illustrated above related to the selection of the best parameter sets.

**Fig 12:**
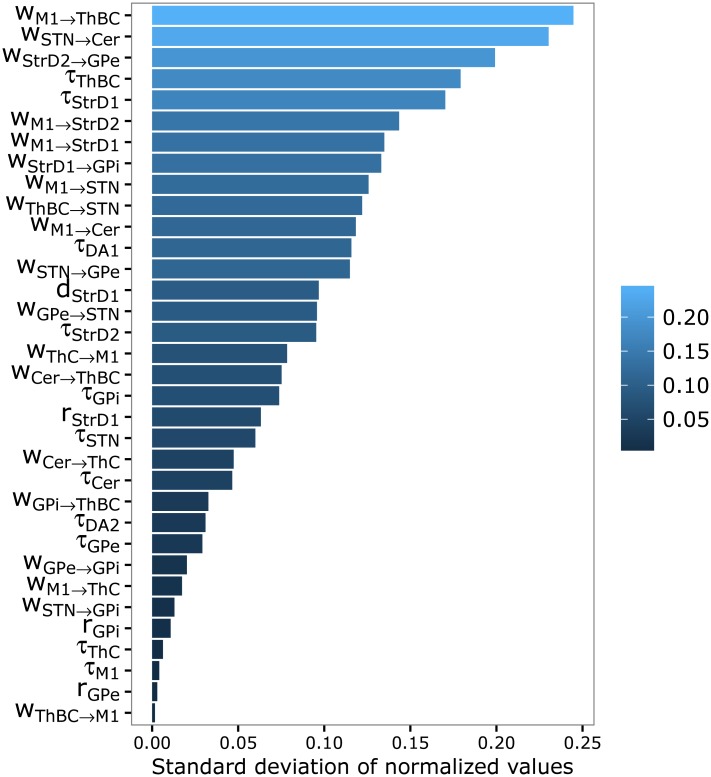
Standard deviation, ranked in decreasing order of magnitude, of some of the model parameters sets found with the genetic algorithm optimisation procedure. The parameter sets used to compute the standard deviation were those having a data fitting error within the first quartile. Abbreviations of neural areas are summarized in S1 Table as well as in the caption of Fig 2. The other symbols used in the figure are as follows: *r*: unit resting potential (Eq 3); *τ*: unit decay coefficient (Eq 1); *w*_*pre* → *post*_: connection weight connecting a unit of the pre component to a unit of the post component (Eq 3); *d*: dopamine amplitude coefficient (Eqs 4 and 5);

Fig 12 shows the standard deviation of parameters computed with such procedure. The figure shows that the most important parameters to ensure a good fit of the target data by the model are those involving the ThBC efferent connections reaching M1 (*w*_*ThBC* → *M*1_): this indicates that ThBC might be important as it integrates information from BG (dishinibition) and from Cer (activation) and we have seen that a concurrent activation of BG and M1, supported by Cer, is important for the production of tics. In this respect, note the lesser importance of the Th reached only by Cer (*w*_*ThC* → *M*1_). The STN efferent connections reaching GPi (*w*_*STN* → *GPi*_), GPi parameters (*r*_*GPi*_) and other parameters related to the indirect pathway (*w*_*GPe* → *GPi*_, *r*_*GPe*_) are also very important, stressing the relevance of BG activation to trigger tics. The efferent connections of M1 towards the ThC (*w*_*M*1 → *ThC*_), Cer (*w*_*M*1 → *Cer*_) and BG (*w*_*M*1 → *STN*_), as well as those from the Cer towards Th (*w*_*Cer* → *ThBC*_, *w*_*Cer* → *ThC*_), have a medium importance. At the opposite side of the spectrum, we find the parameters related to the efferent connections of M1 towards the ThBC (*w*_*M*1 → *ThBC*_) and towards Dorsal putamen (*w*_*M*1 → *StrD*2_, *w*_*M*1 → *StrD*1_) and the connection linking BG to Cer (*w*_*STN* → *Cer*_).

## Discussion

McCairn and colleagues [49] presented an animal model suggesting that tics are produced by dynamical processes involving a complex brain system formed by at least basal ganglia and cortex. The results produced with the model indicate that dopamine bursts represent a necessary condition for the motor tic production. However, these bursts alone are not sufficient for tic generation as in some cases abnormal dopaminergic bursts take place without a co-occurring tic generation. The model presents an operational hypothesis on how the interplay of basal ganglia and cortex (see Fig 2) leads to tic production (see Fig 9). The hypothesis proposes that tic production pivots on the typical selection processes implemented by the basal ganglia-thalamo-cortical loops. These processes select intrinsic noise and inputs received by the system from other cortical regions. Specifically, within the basal ganglia the interplay of the direct, indirect, and hyper-direct pathways (see section “The basal ganglia and their loops with the thalamo-cortical system: anatomy and physiology”) allow such signals to tend to disinhibit the activation of the primary motor cortex. An abnormal dopamine efflux makes the selection mechanisms overly sensitive to the received signals so that even spurious primary motor cortex activations, *if coincident in time*, are actually disinhibited. These activations are then amplified by the thalamo-cortical circuit and as a consequence an overt tic motor movement is released.

The proposed hypothesis agrees with evidence showing that an alteration in striatal phasic dopamine release may underlie the generation of tics as in Tourette syndrome as a consequence of an abnormal focal excitation within the striatum that causes a maladaptive disinhibitory action of basal ganglia. This disinhibition, in turn, may release an abnormal activation of cortical neurons [15, 17] resulting in the production of tics.

McCairn and colleagues [49] suggest that the aberrant activity in the cerebellar cortex during tics production might be influenced by the disynaptic link connecting the basal ganglia to the cerebellum [30]. The authors, however, only mentioned this hypothesis without specifying the neural mechanisms underlying the basal ganglia-cerebellar abnormal interaction. The model proposed here supports the computational feasibility of the intuition of McCairn and colleagues suggesting a specific operational hypothesis on how this might happen. In particular, the model proposes that when tics are produced, the activity in the subthalamic nucleus increases, and this affects the cerebellum activity through the disynaptic link. The increase of the activity in the cerebellum as a consequence of the increased activity in the subthalamic nucleus reproduced by the model agrees with anatomical evidence showing that the output neurons of the subthalamic nucleus are excitatory/glutamatergic and they project to the cerebellum through the pontine nuclei whose output neurons to the cerebellum are largely glutamatergic [30, 91, 133]. Moreover, the cerebellum activation by the subthalamic nucleus is indirectly supported by several empirical experiments (e.g., [43, 134]). Once activated, the cerebellum might feedback to the basal-ganglia and also directly affect the descending motor pathways: these processes, not investigated here, are further discussed below.

The system-level neural mechanisms for tic production suggested by the model yield some predictions that could be tested in future empirical experiments. In particular, the model predicts that the number of tics increases with dopamine (Fig 11). This increase may be due to a further increase of the sensitivity of the selection process within the basal ganglia-thalamo-cortical system. This implies that spurious activations of primary motor cortex may be selected more easily and develop into motor tics (cf., Fig 9). This result could support the design of future therapeutic actions based on dopaminergic modulation [135]. In addition, the model predicts that reducing the spurious activity of primary motor cortex might reduce tic events (Fig 9). This suggests the possibility of developing non-invasive therapeutic interventions targeting either thalamo-cortical loops or primary motor cortex (e.g., through tDCS or transcranial magnetic stimulation (TMS), [6, 136–138]) to possibly affect the plasticity mechanisms happening therein and reduce undesired activations. Similar external manipulations could also be applied to the cerebellum, given that it can help amplify overt tic movements (e.g., abnormal eye blinking) through the dentate projections to the lateral and medial descending systems for motor execution, and to the vestibular nuclei for balance and eye control (Fig 2) [117].

### Conclusions and future work

This work proposes a computational system-level model that reproduces the recent data obtained in [49], proposes a detailed hypothesis of the brain mechanisms that might possibly underlie them, and produces predictions that point to new brain areas as targets for future therapeutic interventions. In particular, the model furnishes an explanation of the neural mechanisms underlying Tourette syndrome that pivots on integrated basal ganglia-thalamo-cortical action selection processes and on the recently discovered subthalamic nucleus-pons-cerebellar connection [29, 30].

Notwithstanding its novelty, the model has relevant limitations that represent starting points for future research. First, future versions of the model could include more detailed versions of thalamus and cortex (e.g., [76, 77, 139, 140]) in order to study more in detail the mechanisms through which such brain components contribute to tic production. Indeed, the dynamics of the thalamo-cortical subsystem are very important for the production of motor movements [72], and so their better understanding might also be important for a better view of the production of dysfuntional motor tics. Similarly, future versions of the model could also study the effects of dopamine in the subthalamic nucleus in Tourette syndrome [141] as well as the results of recent data on the role of the nucleus accumbens and the related limbic network in tic generation [142].

Second, future research could investigate how the increased cerebellar activation by the subthalamic nucleus could modulate the tic intensity through the cerebello-thalamo-cortical circuit. This view is in line with previous theoretical proposals [46, 65, 66] and empirical evidence [37, 143] highlighting the role of the basal ganglia for triggering movements and of the cerebellum for motor pattern amplification. Empirical evidence indicates that an increased activation of the cerebellum tends to cause an increased activity to the primary motor cortex through the thalamo-cortical pathway [44, 144]. Moreover, evidence also indicates that cerebellar hyperactivity may indeed contribute to tic production in Tourette syndrome patients [48, 145].

Third, the model could be modified to reproduce the dopamine-based learning processes of basal ganglia, [146], and also the inferior olive-climbing fibers circuit believed to provide error signals to the cerebellar cortex [147], to investigate how these plasticity events may affect tic emergence in Tourette syndrome. In this respect, the model could be used to address data suggesting that unmedicated individuals with Tourette syndrome learn better from rewards than from punishments [148, 149]. Along the same line, the model could be used to study recent findings suggesting that the involuntary and recurrent nature of tics could be a manifestation of overlearned motor patterns due to excessive LTP in the cerebellar cortex [147], an hypothesis supported by the fact that such abnormal learning processes can be interrupted by modulation of cerebellar activity through non-invasive brain stimulation [150].

Lastly, the model proposes one possible role of the subthalamic-pons-cerebellar circuit [29, 30]. The discovery of these connections has raised fundamental questions on how basal ganglia and cerebellum might directly influence each other [35, 55]. In this respect, the model represents the first computational proposal suggesting a possible role of the basal ganglia-cerebellum connection, in particular assigning to the cerebellum a role in tic production. However, alternative possible roles based on the literature should be compared to this one. For example, the subthalamic nucleus is part of the indirect pathway of the basal ganglia and is implicated in action inhibition and aversive learning [151, 152]. Thus, another possible role of the subthalamic-pons-cerebellar circuit might be to provide a stop signal to the cerebellum for withholding ongoing movements [45]. Another possibility might be that the subthalamic nucleus signals the cerebellum an “off-line” status of the system, in particular that the subthalamic nucleus itself is withholding motor programs via the excitation of globus pallidus and substantia nigra reticulata in turn inhibiting respectively the thalamus and midbrain motor nuclei. The purpose of this would be to allow the cerebellar internal models to be safely used for off-line mental simulation [45, 153, 154]. Further research will be necessary to understand the functions of the newly discovered pathway [35, 55].

Notwithstanding the need for these further studies, we think the model offers a system-level framework supporting our understanding of the brain mechanisms underlying tic production. This framework is expected to support an increasingly integrated interpretation of existing data, and also the design of novel empirical experiments and therapeutic interventions under the guidance of a wider systemic perspective.

## Supporting information

S1 Table Abbreviations used in the article. (EPS)

S2 Table Values of the parameters of the model. The star indicates the values obtained with the genetic algorithm. Abbreviations of neural areas are summarized in S1 Table as well as in the caption of Fig 2. The other symbols used in the table are as follows: *r*: unit resting potential (Eq 3); *τ*: unit decay coefficient (Eq 1); *w*_*pre* → *post*_: connection weight connecting a unit of the pre component to a unit of the post component (Eq 3); *w*_*StrInh*_: inner inhibitory connection weight of striatal regions; *SD*_*BG*1_: standard deviation of the Gaussian noise signal affecting StrD1, StrD2, and STN (Eq 3); *SD*_*BG*2_: standard deviation of the Gaussian noise signal affecting GPe, GPi (Eq 3); *Mean*_*BG*_: mean of the Gaussian noise signal affecting all BG sub-components (Eq 3); *SD*_*Th*_ and *Mean*_*Th*_: standard deviation and mean of the Gaussian noise signal affecting ThBC and ThC (Eq 3); *SD*_*MF*_ and *Mean*_*MF*_: standard deviation and mean of the Gaussian noise signal affecting MF (Eq 3); *SD*_*M*1_ and *Mean*_*M*1_: standard deviation and mean of the Gaussian noise signal affecting M1 (Eq 3); b: baseline coefficient (Eqs 4 and 5); d: dopamine amplitude coefficient (Eqs 4 and 5)
(EPS)

S3 Table Average value of the peak amplitude of the activity (mean) and standard deviation (SD) for several areas of the model and for each movement state (NO-TIC vs TIC). (EPS)
